# Molecular Discrimination of Sheep Bovine Spongiform Encephalopathy from Scrapie

**DOI:** 10.3201/eid1704.101215

**Published:** 2011-04

**Authors:** Laura Pirisinu, Sergio Migliore, Michele Angelo Di Bari, Elena Esposito, Thierry Baron, Claudia D’Agostino, Luigi De Grossi, Gabriele Vaccari, Umberto Agrimi, Romolo Nonno

**Affiliations:** Author affiliations: Istituto Superiore di Sanità, Rome, Italy (L. Pirisinu, S. Migliore, M.A. Di Bari, E. Esposito, C. D’Agostino, G. Vaccari, U. Agrimi, R. Nonno);; Agence Nationale de Sécurité Sanitaire, Lyon, France (T. Baron);; Istituto Zooprofilattico Sperimentale delle Regioni Lazio e Toscana, Rome (L. De Grossi)

**Keywords:** Prions, transmissible spongiform encephalopathy, scrapie, bovine spongiform encephalopathy, BSE, CH1641, sheep, Western blot, dispatch

## Abstract

Sheep CH1641-like transmissible spongiform encephalopathy isolates have shown molecular similarities to bovine spongiform encephalopathy (BSE) isolates. We report that the prion protein PrP^Sc^ from sheep BSE is extremely resistant to denaturation. This feature, combined with the N-terminal PrP^Sc^ cleavage, allowed differentiation of classical scrapie, including CH1641-like**,** from natural goat BSE and experimental sheep BSE.

Prion diseases, or transmissible spongiform encephalopathies (TSEs), are neurodegenerative disorders that include Creutzfeldt-Jakob disease (CJD) in humans, scrapie in sheep and goats, and bovine spongiform encephalopathy (BSE) in cattle. TSEs are characterized by accumulation of an abnormal isoform of the host-encoded prion protein (PrP^C^), termed PrP^Sc^.

A novel human prion disease, variant CJD, was reported in 1995 and postulated to be caused by eating beef infected with BSE. Biologic and molecular analyses provided evidence that the same agent was involved in BSE and variant CJD (*1**,**2*). Evidence of sheep and goat susceptibility to BSE (*3*) and discovery of natural BSE infections in 2 goats (*4**,**5*) prompted the European Commission to increase the search for BSE infections in small ruminants. Although the BSE agent can be recognized by biologic strain typing in conventional mice (*2*), large-scale testing of small ruminants required molecular tests able to discriminate BSE from the most common TSEs of small ruminants.

Molecular criteria used to discriminate BSE from scrapie are based on the low molecular weight of proteinase K–treated PrP^Sc^ (PrP^res^) (*6**–**8*), a high proportion of the diglycosylated PrP^Sc^ (*1**,**6**,**8*), and poor or absent binding with antibodies directed at N-terminal epitopes (*8**–**10*). This last characteristic was fundamental in developing the discriminatory methods currently approved for surveillance in Europe (*11*).

The experimental scrapie isolate CH1641 reportedly shares molecular features with experimental sheep BSE (*7*), although lack of transmissibility of CH1641 to conventional mice in comparison to successful transmission of BSE provided evidence that CH1641 and BSE are caused by distinct prion agents. A few natural isolates have been described in sheep, showing molecular (*10**,**12*) and biologic (*13*) similarities to CH1641, and were named CH1641-like. Subtle pathologic differences were exploited to distinguish these CH1641-like isolates from BSE by immunohistochemical (*5**,**10*) and biochemical analyses by glycoform profiling (*8**,**10*). However, routine testing by using discriminatory Western blot (WB) methods does not easily distinguish CH1641 and CH1641-like isolates from BSE (*8**,**12*). We report 2 new CH1641-like isolates; analyze the conformational stability of CH1641-like isolates, BSE, and classical scrapie; and show that a reliable molecular differentiation of these 3 TSE sources is possible by an improved discriminatory WB method.

## The Study

During 2009–2010, we analyzed conformational stability of PrP^Sc^ from sheep TSE isolates by using a conformational stability and solubility assay (CSSA) that we developed (*14*). We showed that CSSA could reveal strain-specified PrP^Sc^ conformational stability in sheep isolates because it enabled discrimination of Nor98 from classical scrapie isolates (*14*). Scrapie isolates had GdnHCl_1/2_ values (the concentration of guanidine hydrochloride able to dissolve half the insoluble PrP^Sc^ aggregates in a brain homogenate) of 2.0 mol/L–2.3 mol/L; Nor98 isolates were less stable (1.3–1.4 mol/L GdnHCl). We thus sought to determine the conformational stability of PrP^Sc^ aggregates (Technical Appendix) derived from CH1641 and BSE strains (Table 1), including one (TR316211) of the few CH1641-like field isolates described so far (*10**,**12**,**13*). Two other CH1641-like isolates (99–454 and 99–321) were found in a retrospective analysis of sheep scrapie cases in France.

**Table 1:** Transmissible spongiform encephalopathy isolates analyzed by conformational stability and solubility assay*

Source	Identification no.	PrP genotype†	GdnHCl_1/2_, mol/L ± SD‡
Natural isolates			
Scrapie	ES/8/10/2	ARQ/ARQ	2.19 ± 0.18
CH1641-like	99–454	VRQ/VRQ	2.00 ± 0.06
	99–321	VRQ/VRQ	2.41 ± 0.49
	TR316211	ARQ/ARQ	2.82 ± 0.08
Experimental samples			
CH1641	241/74	AxQ/AxQ	2.07 ± 0.05
Sheep BSE	301/16§	ARQ/ARQ	>4
	301/44§	ARQ/ARQ	>4
	302/90¶	ARQ/ARQ	3.8; >4; >4

*PrP, prion protein; GdnHCL_1/2_, guanidine hydrochloride at a concentration able to dissolve half the insoluble aggregates in a brain homogenate; BSE, bovine spongiform encephalopathy. †Amino acids at codons 136, 154 and 171. ‡Each sample was analyzed >3 times. §Intracerebral transmission. ¶Oral transmission.

Classical scrapie included as control displayed a GdnHCl_1/2_ value (2.2 mol/L) in the range of previously analyzed isolates. CH1641 (provided by N. Hunter, Institute for Animal Health, Edinburgh, Scotland) and CH1641-like isolates showed conformational stabilities close to classical scrapie, with GdnHCl_1/2_ values of 2.0–2.8 mol/L. In contrast, PrP^Sc^ from experimental sheep BSE (*15*) clearly showed higher conformational stability, with GdnHCl_1/2_ values >3.8 mol/L (Table 1). These results suggest experimental sheep BSE might have a stronger resistance to denaturation than do most natural sheep scrapie isolates.

**Figure 1:**
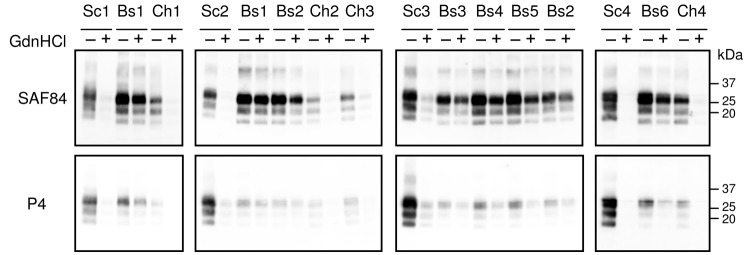
Representative Western blot showing the differential N-terminal proteinase K cleavage (monoclonal antibodies SAF84 vs. P4) and the susceptibility to denaturation of different transmissible spongiform encephalopathy isolates. Samples are indicated according to Table 2: classical scrapie isolates (Sc1, Sc2, Sc3, Sc4); experimental CH1641 (Ch1); CH1641-like isolates (Ch2, Ch3, Ch4); experimental sheep bovine spongiform encephalopathy by intracerebral transmission (Bs1) and oral transmission (Bs2, Bs3, Bs4, Bs5); natural goat isolate (Bs6). All samples were pretreated (+) or not treated (-) with 3.5 mol/L guanidine hydrochloride for 1 h at 37°C and then diluted to a final concentration of 0.35 mol/L guanidine hydrochloride, before digestion with proteinase K according to the Istituto Superiore di Sanità discriminatory method. Replica blots were probed with SAF84 (top) and P4 (bottom). Molecular weights are indicated on the right. GdnHCl, guanidine hydrochloride.

Because the discriminatory methods based on differential PrP^Sc^ N-terminal proteinase K (PK) cleavage (*11*) do not enable a clear-cut discrimination of CH1641-like from BSE (*12*), we investigated the potential of denaturation with GdnHCl as a further discriminatory strategy within the framework of the Istituto Superiore di Sanità discriminatory WB (*11*). To this aim, samples were untreated or treated with 3.5 mol/L GdnHCl before PK digestion and WB analysis with SAF84 and P4 monoclonal antibodies (Figure 1). This method was set up by analyzing representative scrapie, BSE, and CH1641 samples (Figure 1). As expected, BSE and CH1641 were poorly detected by P4, in contrast to classical scrapie. Treatment with 3.5 M GdnHCl, however, nearly abolished PK resistance of PrP^Sc^ from classical scrapie and CH1641, but not from sheep BSE, thus also enabling discrimination of CH1641 from BSE.

**Figure 2:**
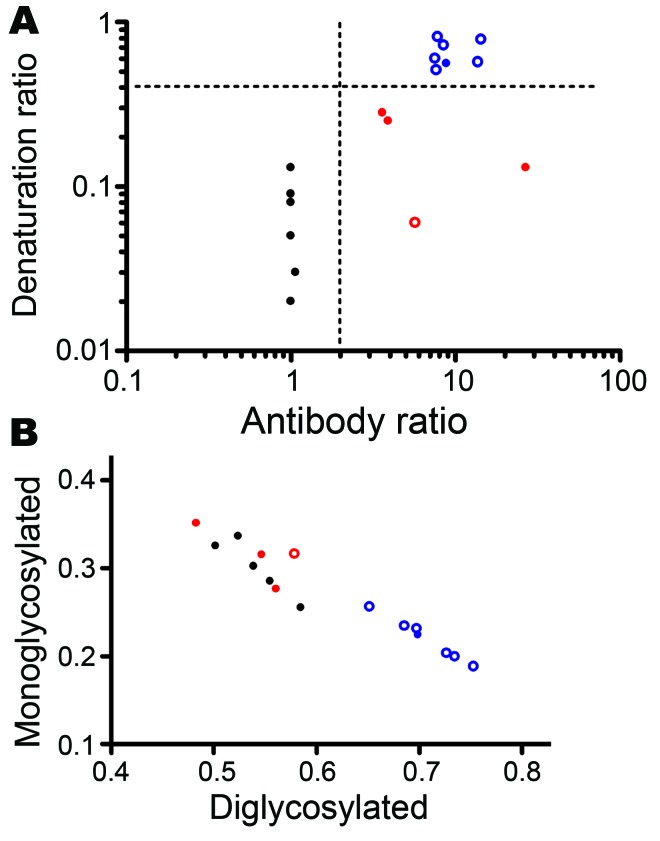
A) Scattergraph of antibody ratio and denaturation ratio obtained from each sample in Table 2, showing discrimination of scrapie, CH1641, CH1641-like, and bovine spongiform encephalopathy (BSE) samples. The antibody ratio is the SAF84/P4 ratio of the chemiluminescence signal relative to the SAF84/P4 ratio of the control scrapie loaded in each blot (Technical Appendix). The denaturation ratio, obtained from the SAF84 blot, is the ratio between the chemiluminescence signal with 3.5 mol/L and that with 0 mol/L. The vertical dashed line refers to the cutoff value of the antibody ratio, according to the Istituto Superiore di Sanità discriminatory Western blot (antibody ratio 2). The horizontal dashed line (denaturation ratio 0.4) shows the separation of BSE samples from all other transmissible spongiform encephalopathy sources. B) Scattergraph of proportions of diglycosylated and monoglycosylated PrPres bands from samples in Table 2. Results were obtained from guanidine hydrochloride–untreated samples in blots treated with SAF84. Classical scrapie samples are represented by black symbols, CH1641 by red symbols, and BSE samples by blue symbols. Filled symbols denote natural isolates and open symbols represent the experimental isolates.

We then analyzed a larger set of samples (Table 2), including natural BSE in a goat (Figure 1). These experiments confirmed the higher resistance to denaturation of BSE samples, irrespective of the species, PrP genotype, and route of inoculation, compared with all other samples (Figure 1). When the antibody ratio and the denaturation ratio were measured and plotted as a scattergraph, classical scrapie, CH1641, and BSE isolates clustered into 3 distinct groups (Figure 2, panel A): 1) scrapie isolates displayed antibody ratios <2 and denaturation ratios were 0.02–0.13; 2) CH1641 samples had antibody ratios >2 and denaturation ratios were 0.06–0.29; and 3) BSE samples had antibody ratios >2, but denaturation ratios were >0.51.

**Table 2:** Transmissible spongiform encephalopathy samples analyzed by discriminatory Western blot*

Source	Identification no.	PrP genotype†	Blot lane
Natural isolates			
Scrapie	ES16/10/10	ARQ/ARQ	Sc1
	ES16/10/11	ARQ/ARQ	Sc2
	ES16/10/12	ARQ/ARQ	Sc3
	ES12/10/1	ARQ/ARQ	
	ES12/10/2	ARQ/ARQ	
	ES12/10/3	ARQ/ARQ	Sc4
CH1641-like	99–454	VRQ/VRQ	Ch2
	99–321	VRQ/VRQ	Ch4
	TR316211	ARQ/ARQ	Ch3
Goat BSE	CH636		Bs6
Experimental samples			
CH1641	241/74	AxQ/AxQ	Ch1
Sheep BSE	301/16‡	ARQ/ARQ	Bs1
	301/44‡	ARQ/ARQ	
	302/87§	ARQ/ARQ	Bs3
	302/130§	ARQ/ARQ	Bs4
	302/64§	ARQ/AHQ	Bs5
	302/90§	ARQ/ARQ	Bs2

*Blot lanes shown in Figure 1. PrP, prion protein; BSE, bovine spongiform encephalopathy. †Amino acids at codons 136, 154 and 171. ‡Intracerebral transmission. §Oral transmission.

Glycoform profiles, i.e., the relative proportion of diglycosylated, monoglycosylated, and unglycosylated PrP^res^ fragments, have also been reported as a discriminatory criterion for the identification of BSE in sheep (*8**–**10*), as well as when compared with CH1641 (*8**,**10*). With the Istituto Superiore di Sanità WB method (Figure 2, panel B), field scrapie isolates, including CH1641-like isolates, were characterized by a lower diglycosylated-to-monoglycosylated glycoform ratio (0.48:0.35–0.58:0.25) than sheep BSE (0.65:0.25–0.75:0.19) and the natural goat BSE (0.70:0.22).

## Conclusions

Because the analysis of PrP^Sc^ from sheep prion isolates by CSSA showed an extremely high conformational stability of BSE samples, we improved the Istituto Superiore di Sanità discriminatory WB by including a pretreatment of brain homogenates with GdnHCl. Our results show that the combined use of 2 independent molecular features, N-terminal cleavage by PK and resistance to denaturation, could indeed differentiate classical scrapie and CH1641-like isolates from small ruminant BSE. Nonetheless, we observed some variability among the CH1641-like samples, either when analyzed by CSSA (Table 1) or by the discriminatory WB. As previously reported (*12*), the antibody ratios of some CH1641-like samples were close to the cutoff (Figure 2, panel A). Furthermore, the variable conformational stability observed by CSSA was also reflected in the denaturation ratios measured by discriminatory WB, with 2 CH1641-like samples showing a relatively higher resistance to GdnHCl than to all other scrapie samples. Because of the limited number of CH1641-like isolates, further studies are needed to evaluate their effective range of variability.

This variability may be disappointing for discriminatory purposes, but it may also hinder the possible presence of subtle PrP^Sc^ conformational (and possibly strain) variants in CH1641-like isolates. The biologic similarities of CH1641-like samples after transmission to ovine transgenic mice (*13*) and voles (U. Agrimi, unpub. data) were worth noting. Nevertheless, CH1641-like isolates induced a certain degree of PrP^Sc^ molecular variability in both rodent models (*13*; U. Agrimi, unpub. data), which might be related to the molecular variability in PrP^Sc^ extracted from sheep brain.

Although based on a limited set of samples, our study supports the notion that CH1641-like isolates can be convincingly discriminated from small ruminant BSE on molecular grounds. Furthermore, the high conformational stability of BSE, when compared with that in classical scrapie, Nor98, and CH1641-like isolates, suggests the potential of the new discriminatory WB here proposed for discriminating BSE from other known small ruminant TSEs.

## Supplementary Material

Technical Appendix Provides additional information on the Determination of the SAF84/P4 antibody ratio.

## References

[1] Collinge J, Sidle KC, Meads J, Ironside J, Hill AF. Molecular analysis of prion strain variation and the aetiology of ‘new variant’ CJD. Nature. 1996;383:685–90. 10.1038/383685a0 8878476

[2] Bruce ME, Will RG, Ironside JW, McConnell I, Drummond D, Suttie A, Transmissions to mice indicate that ‘new variant’ CJD is caused by the BSE agent. Nature. 1997;389:498–501. 10.1038/39057 9333239

[3] Foster JD, Hope J, Fraser H. Transmission of bovine spongiform encephalopathy to sheep and goats. Vet Rec. 1993;133:339–41. 10.1136/vr.133.14.339 8236676

[4] Eloit M, Adjou K, Coulpier M, Fontaine JJ, Hamel R, Lilin T, BSE agent signatures in a goat. Vet Rec. 2005;156:523–4. 15833975 10.1136/vr.156.16.523-b

[5] Jeffrey M, Martin S, Gonzalez L, Foster J, Langeveld JP, van Zijderveld FG, Immunohistochemical features of PrP(d) accumulation in natural and experimental goat transmissible spongiform encephalopathies. J Comp Pathol. 2006;134:171–81. 10.1016/j.jcpa.2005.10.003 16542672

[6] Hill AF, Sidle KC, Joiner S, Keyes P, Martin TC, Dawson M, Molecular screening of sheep for bovine spongiform encephalopathy. Neurosci Lett. 1998;255:159–62. 10.1016/S0304-3940(98)00736-8 9832197

[7] Baron TG, Madec JY, Calavas D, Richard Y, Barillet F. Comparison of French natural scrapie isolates with bovine spongiform encephalopathy and experimental scrapie infected sheep. Neurosci Lett. 2000;284:175–8. 10.1016/S0304-3940(00)01047-8 10773427

[8] Stack MJ, Chaplin MJ, Clark J. Differentiation of prion protein glycoforms from naturally occurring sheep scrapie, sheep-passaged scrapie strains (CH1641 and SSBP1), bovine spongiform encephalopathy (BSE) cases and Romney and Cheviot breed sheep experimentally inoculated with BSE using two monoclonal antibodies. Acta Neuropathol. 2002;104:279–86. 12172914 10.1007/s00401-002-0556-2

[9] Thuring CM, Erkens JH, Jacobs JG, Bossers A, Van Keulen LJ, Garssen GJ, Discrimination between scrapie and bovine spongiform encephalopathy in sheep by molecular size, immunoreactivity, and glycoprofile of prion protein. J Clin Microbiol. 2004;42:972–80. 10.1128/JCM.42.3.972-980.2004 15004040 PMC356877

[10] Lezmi S, Martin S, Simon S, Comoy E, Bencsik A, Deslys JP, Comparative molecular analysis of the abnormal prion protein in field scrapie cases and experimental bovine spongiform encephalopathy in sheep by use of Western blotting and immunohistochemical methods. J Virol. 2004;78:3654–62. 10.1128/JVI.78.7.3654-3662.2004 15016886 PMC371064

[11] Community Reference Laboratory of the European Union. TSE strain characterization in small ruminants—a technical handbook for national reference laboratories in the EU. Version 4, January 2010 [cited 2011 Feb 14]. http://www.defra.gov.uk/vla/science/docs/sci_tse_rl_handbookvjan10.pdf

[12] Stack M, Jeffrey M, Gubbins S, Grimmer S, Gonzalez L, Martin S, Monitoring for bovine spongiform encephalopathy in sheep in Great Britain, 1998–2004. J Gen Virol. 2006;87:2099–107. 10.1099/vir.0.81254-0 16760414

[13] Baron T, Bencsik A, Vulin J, Biacabe AG, Morignat E, Verchere J, A C-terminal protease-resistant prion fragment distinguishes ovine “CH1641-like” scrapie from bovine classical and L-type BSE in ovine transgenic mice. PLoS Pathog. 2008;4:e1000137. 10.1371/journal.ppat.1000137 18769714 PMC2516186

[14] Pirisinu L, Di Bari M, Marcon S, Vaccari G, D’Agostino C, Fazzi P, A new method for the characterization of strain-specific conformational stability of protease-sensitive and protease-resistant PrP^Sc^. PLoS ONE. 2010;5:e12723. 10.1371/journal.pone.0012723 20856860 PMC2939050

[15] Vaccari G, D’Agostino C, Nonno R, Rosone F, Conte M, Di Bari MA, Prion protein alleles showing a protective effect on the susceptibility of sheep to scrapie and bovine spongiform encephalopathy. J Virol. 2007;81:7306–9. 10.1128/JVI.02880-06 17442723 PMC1933282

