# Supplementary material for: Molecular Discrimination of Sheep Bovine Spongiform Encephalopathy from Scrapie
Source: Emerg Infect Dis. 2011 Apr;17(4):695–8. doi: 10.3201/eid1704.101215 (PMC3377410; doi:10.3201/eid1704.101215)
Supplement: Technical Appendix — Provides additional information on the Determination of the SAF84/P4 antibody ratio. [file 10-1215-Techapp.pdf]

Article DOI: 10.3201/eid1704.101215

Molecular Discrimination of Sheep Bovine Spongiform Encephalopathy from Scrapie

Technical Appendix

The conformational stability and solubility assay uses denaturation with increasing concentrations of guanidine hydrochloride (GdnHCl) followed by differential centrifugation and eanbles determination of the concentration that can dissolve half the insoluble abnormal isoform of host-encoded prion protein (PrP^{Sc}) aggregates present in a brain homogenate ($GdnHCl_{1/2}$). Aliquots of brain homogenates (3%–6% wt/vol) were incubated for 1 h at 37°C with gentle shaking in 100 mmol/L Tris-HCl (pH 7.4) containing 2% sarcosyl and then treated with GdnHCl solutions for 1 h at 37°C with gentle shaking, with final GdnHCl concentrations ranging from 0 mol/L to 4.0 mol/LM. Samples were then subjected to differential centrifugation (20,000 × *g* for 1 h at 22°C) which enables separation of insoluble PrP^{Sc} aggregates from most of the soluble PrP^{C} present in the brain homogenate. Pellets were resuspended in 90 µL NuPage LDS Sample Buffer (Invitrogen, Carlsbad, CA, USA) and 10 µL NuPage Sample Reducing Agent (Invitrogen) and analyzed by Western blotting with monoclonal antibody SAF84. Chemiluminescence signal was detected with the VersaDoc imaging system (Bio-Rad, Hercules, CA, USA) and was quantified by using QuantityOne software (Bio-Rad). Individual denaturation curves were analyzed and best-fitted by plotting the fraction of PrP^{Sc} remaining in the pellet as a function of GdnHCl concentration, and using a 4-parameter logistic equation (GraphPad Prism; Graphpad Software Inc., La Jolla, CA, USA).

Determination of the SAF84/P4 antibody ratio

The antibody ratio is the ratio of the chemiluminescence signal, produced by a given sample when determined separately with SAF84 and P4 monoclonal antibodies, relative to the SAF84/P4 ratio of the control scrapie. This ratio measure the cleavage of the N-terminal P4 epitope of PrP^{Sc}, which occurs in sheep bovine spongiform encephalopathy but not in most scrapie cases. To obtain the relative SAF84/P4 ratio, we calculate the absolute ratio of SAF84/P4

volumes for each sample and the scrapie control and then divide the absolute ratio of each sample by the absolute ratio of the scrapie control. (Istituto Superiore di Sanità discriminatory Western blot; Community Reference Laboratory of the European Union: TSE strain characterization in small ruminants—a technical handbook for national reference laboratories in the EU. 174 Version 4, January 2010 [cited 2011 Feb 14]. http://www.defra.gov.uk/vla/science/docs/sci_tse_rl_handbookv4jan10.pdf).
